# Systematic gene silencing identified *Cryptosporidium* nucleoside diphosphate kinase and other molecules as targets for suppression of parasite proliferation in human intestinal cells

**DOI:** 10.1038/s41598-019-48544-z

**Published:** 2019-08-21

**Authors:** A. Castellanos-Gonzalez, G. Martinez-Traverso, K. Fishbeck, S. Nava, A. C. White

**Affiliations:** Infectious Disease Division, Department of Internal Medicine, University of Texas Medical Branch, Galveston, TX USA

**Keywords:** Microbiology, Gastrointestinal diseases

## Abstract

Cryptosporidiosis is a major cause of diarrheal disease. The only drug approved for cryptosporidiosis has limited efficacy in high-risk populations. Therefore novel drugs are urgently needed. We have identified several enzymes as potential targets for drug development and we have optimized a rapid method to silence genes in *Cryptosporidium*. In this study, we knocked down expression of the four selected genes: Actin (Act), Apicomplexan DNA-binding protein (Ap2), Rhomboid protein 1 (Rom 1), and nucleoside diphosphate kinase (NDK). After gene silencing, we evaluated the role of each target on parasite development using *in vitro* models of excystation, invasion, proliferation, and egress. We showed that silencing of Act, Ap2, NDK, and Rom1 reduced invasion, proliferation, and egress of *Cryptosporidium*. However, silencing of NDK markedly inhibited *Cryptosporidium* proliferation (~70%). We used an infection model to evaluate the anticryptosporidial activity of ellagic acid (EA), an NDK inhibitor. We showed that EA (EC50 = 15–30 µM) reduced parasite burden without showing human cell toxicity. Here, we demonstrated the usefulness of a rapid silencing method to identify novel targets for drug development. Because EA is a dietary supplement already approved for human use, this compound should be studied as a potential treatment for cryptosporidiosis.

## Introduction

*Cryptosporidium* is a leading cause of moderate-to-severe diarrhoea in children <2 years of age and the most common pathogen associated with death in toddlers (age range, 12–23 months^1^). Nitazoxanide is the only FDA-approved medication available for cryptosporidiosis treatment, but it has limited efficacy when used to treat those at the highest risks for poor outcomes. More effective treatment options are urgently needed^2–4^. The limitations of tools used to genetically manipulate gene expression in this parasite have been identified as a major hurdle for drug and vaccine development^2,4^. We developed a method to silence genes in this parasite using pre-assembled complexes of *Cryptosporidium* single-stranded RNA (ssRNA) and the human enzyme Argonaute 2 (hAgo2)^5^. In this method, ssRNA is loaded into the enzyme hAgo2 to form a minimal RNA-induced silencing complex (RISC)^6^, after parasite transfection, the antisense ssRNA guides the complex to the mRNA target, by binding, gene expression is blocked and mRNA is degraded by the slicer activity of Ago2 producing the silencing. We hypothesised that this method can be used to determine the role of *Cryptosporidium* genes expressed during the infection of human cells, therefore could be useful to identify novel targets for drug and vaccine development. In this study, we evaluate the role of four genes potently silenced (>70%) with this method: Actin (Act), Rhomboid protein 1 (Rom1), transcription factor Ap2 (Ap2), and nucleoside diphosphate kinase (NDK) 1. In addition we tested the anticryptosporial activity of the ellagic acid (an NDK inhibitor).

## Methods

### Target selection for silencing experiments

**Figure 1:**
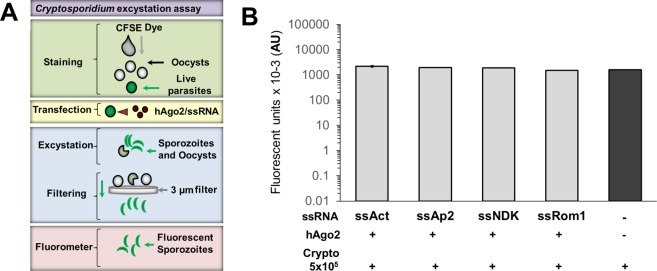
*Cryptosporidium* excystation assay. *Excystation model*. (**A**) Parasites were stained with a vital dye (highlighted in green) and transfected with hAgo2/target ssRNA (highlighted in yellow). Excystation was then induced and sporozoites were filtered (highlighted in blue). Sporozoites were quantified using fluorometry (highlighted in purple). *Effect of silencing on excystation*. (**B**) *Cryptosporidium* oocysts were transfected with silencer complexes (+) or not (−). We induced excystation and compared fluorescence in treated (grey bars) and untreated (black bar) oocysts. Experiments were performed in triplicate. The differences between control and treated samples were not statistically significant.

Initially, we selected 100 genes for silencing experiments (Supplementary Table S1). mRNA sequences were obtained from the CryptoDB (https://cryptodb.org/) and Gene Bank databases (https://www.ncbi.nlm.nih.gov/nucleotide/). Genes were selected based on homology for *Cryptosporidium parvum* and *Cryptosporidium hominis*, with regions that were not homologous to human genes. We also focused on genes that were highly expressed in the early stages of *in vitro* development. The selected genes coded for structural proteins, transcription factors, kinases, or proteases, or some combination of these (Supplementary Table S1). For these genes, we found silencing results ranging from 30–94%. In the current manuscript, we analysed genes that were silenced with >75% efficiency (Table 1, Fig. 1).

**Table 1:** Gene silencing in *Cryptosporidium*.

RT-PCR Amplified Target	Antisense ssRNA 21nt	Ct valute Ago2/ssRNA	Ct value Ago2/unrelated RNA	% silencing ± SD
Actin	ssAct	31.4 ± 0.4	28.3 ± 0.8	78 ± 3
Ap2	ssAp2	25.3 ± 0.3	21.3 ± 0.2	94 ± 01
NDK	ssNDK	27.3 ± 0.7	23.6 ± 0.6	93 ± 01
Rom1	ssRom1	46.5 ± 4.1	43.0 ± 2.7	96 ± 1

Selected targets (yellow column) were silenced using antisense ssRNA (grey column) and hAgo2 complexes. Silencing was evaluated using RT-PCR, and Ct values were determined in cells treated with hAgo2/target ssRNA (red column) and in hAgo2/unrelated ssRNA (green column). The silencing was calculated as fold-change relative to control samples and expressed as percentage values, SD = standard deviation (blue column).

### Antisense ssRNA design

Antisense single-stranded RNA (ssRNA) used in silencing experiments was designed using the computational software sFold 2.2 (http://sfold.wadsworth.org). We used the full sequences of the mRNA targets (accession numbers in Supplementary Table S1) as the template. Initially, we generated all possible ssRNA antisense sequences of 21 nucleotides for each target. However, we only selected the optimal ssRNAs based on sFold ranking. The scores reflected parameters such as local free energy and binding probability (C-G > 40%). We synthesised selected ssRNAs obtained from a commercial vendor (Integrated DNA Technologies, Coralville, IA). For the silencing experiments, the ssRNAs were modified as follows: the 21-nucleotide ssRNAs were capped using phosphorylation at the 5′ end and were modified with a deoxynucleotide tail at the 3′ end (Supplementary Table S2). Scrambled control ssRNA (Supplementary Table S2) was designed using siRNA wizard software (Invivogen, San Diego CA).

### Gene silencing in *Cryptosporidiumparvum* oocysts

For the transfection experiments, we used *Cryptosporidium parvum* oocysts (Iowa isolate) purchased from the University of Arizona (Sterling Laboratories, Tucson, AZ). To prepare them for transfection, the oocysts (1 × 10^6^ for each target) were transferred to 1.5-ml tubes, and each sample was diluted with nuclease-free water (Fisher Scientific, Hampton, NH) and centrifuged for 10 min at a speed of 8,000 rpm (microcentrifuge Eppendorf 5424). The supernatant was discarded, and the pellet was resuspended using 20 µl nuclease-free water. The tubes with the samples were at room temperature before adding transfection reagents. To assemble the silencer complexes, the ssRNAs were first diluted with water to 100 nM, then the samples were heated for 1 min at 95 °C and placed on ice. The ssRNA-hAgo2 complexes were assembled in 1.5-ml microcentrifuge tubes. Each tube contained 2.5 µl diluted 100 nM ssRNA, 2.5 µl (62.5 ng/ul) hAgo2 protein (Sino Biologicals, North Wales, PA), and 15 µl assembling buffer (2 mM Mg(OAc)_2_, 150 mM KOHAc, 30 mM HEPES, 5 mM DTT, nuclease-free water). The mixture was then incubated for 1 h at room temperature. After incubation, the complexes were encapsulated by adding 15 µl protein transfection reagent (PTR) Pro-Ject (Thermo Scientific, Rockford, IL). Each sample was mixed by pipetting and then incubated for 30 min at room temperature. For the transfection experiments, the encapsulated complexes were added to the oocysts and the sample was incubated at room temperature for 2 h. The slicer activity of hAgo2 was activated using incubation at 37 °C for 2 h. The reaction was stopped by adding 350 µl RLT lysis buffer (RNeasy kit, Qiagen, Hilden, Germany), and the samples were stored at −20 °C for posterior analysis using RT-PCR. For some experiments, we used only PTR or PTR with unrelated ssRNAs (scrambled ssRNA; Supplementary Table S2).

### RNA extraction and evaluation of silencing using RT-PCR

Before RNA isolation, each sample (previously stored at −20 °C) was thawed at 95 °C for 2 min. The total RNA was then extracted from each sample using the Qiagen RNeasy Plus Mini Kit (Qiagen, Valencia CA) according to the manufacturer’s instructions. The RNA was eluted from the purification columns using 100 µl RNase-free water, then the concentration of eluted RNA was determined using spectrophotometry (NanoDrop 100 Spectrophotometer; Thermo Fisher Scientific, Waltham MA). The silencing in transfected oocysts was analysed using qRT-PCR and the qScript One-Step SYBR Green qRT-PCR kit, Low ROX (Quanta BioSciences/VWR, Radnor, PA). For the RT-PCR experiments, the reactions were assembled as follows: 2 µl purified RNA template (20 ng/µl), 5 µl One-Step SYBR Green Master Mix, 0.25 μl of each primer at a 10 μM concentration, 0.25 μl qScript One-Step reverse transcriptase, and 4.25 µl nuclease-free water, for a total of 10 μl of mixture per sample. The RT-PCR mixture (total volume, 12 µl) was transferred to 96-well reaction plates (0.1 ml/well) (Applied Biosystems, Foster City, CA), and the RT-PCR amplification was performed using a 7500 Fast Real-Time PCR System (Applied Biosystems, Foster City, CA) and the following cycling conditions: 50 °C for 15 min, 95 °C for 5 min, then 50 cycles of 95 °C for 15 sec and 63 °C for 1 min, followed by a melting point analysis (95 °C for 15 sec, 60 °C for 1 min, 95 °C for 15 sec, and 60 °C for 15 sec). Before fold-change analysis, all the target cycle threshold (Ct) values for each silenced target were normalised against the *Cryptosporidium* GAPDH. We used the ΔΔCt method^7^ to calculate fold-changes between the control samples and the silenced samples. The results were presented using the mean value for each target, with the respective standard deviation values. The primers used for the RT-PCR are presented in Supplementary Table S3.

### Oocyst excystation assays

Excystation of the transfected oocysts was induced using acidic water and taurocholic acid as described. Briefly, the *Cryptosporidium* oocysts were pelleted using centrifugation (500 × *g*), the supernatant was discarded, and the parasites were then resuspended in 25 µl acidic water (pH 2.5) and incubated for 10 min on ice. A total of 250 µl excystation medium (Roswell Park Memorial Institute (RPMI)-1640 medium, 1 × antibiotic/antimycotic solution, and 0.8% taurocholic acid sodium salt hydrate) was then added. The sample was then incubated for 1 h at 37 °C. After excystation, we evaluated the excystation rate and then the sporozoites were stained using the vital dye carboxyfluorescein succinimidyl ester (CFSE) (CellTrace, Thermo Fischer Scientific, Waltham, MA) by adding 2 µM CFSE and incubating the sample in the dark at 37 °C for 15 min. After staining, the sporozoites were separated from the unhatched oocysts by filtration using 3.0-µm pore size nitrocellulose membranes (Merck Millipore Ltd., County Cork, Ireland). To evaluate fluorescence of the filtered samples, 200 µl of each filtered sample was transferred to a 96-well plate (Costar, Corning, NY) and the fluorescence was measured (520 nm) using a microplate reader (FLUOstar Omega, Ortenberg, Germany).

### HCT8 cell culture

We used ileocecal cells (HCT-8 cells, ATCC, Manassas, VA) for the infection experiments. The frozen stock of cells was thawed at 37 °C and then and cultured (in 25 cm flasks) overnight at 37 °C in RPMI-1640 media (Gibco/Thermo Fisher Scientific, Waltham, MA) supplemented with 10% foetal bovine serum (FBS) (Stemcell Technologies, Vancouver, Canada) and 1× antibiotic/antimycotic solution (Gibco/Thermo Fisher Scientific, Waltham, MA. Next day, HCT-8 cells were harvested and cultured (~1 × 10^5^ per well) in 24-well plates (Costar, Corning, NY) at 37 °C for 24 hrs.

### ***In vitro*** invasion assay

Cultured HCT-8 cells were used for the invasion assay. Before infection, transfected parasites were stained and excysted as described. After filtering, approximately 5 × 10^5^ parasites (suspended in 250 µl) were added to HCT-8 cells for 1 h at 37 °C. To quantify non-invading sporozoites, 250 µl supernatant was collected after incubation and fixed using 50 µl 4.2% paraformaldehyde solution (Cytofix/Cytoperm, BD BioSciences, San Jose, CA). To quantify sporozoites adhered to cells, the monolayers were trypsinised by adding 150 µl 0.25% trypsin-EDTA (Gibco/Thermo Fisher Scientific, Waltham, MA) and incubating at 37 °C for 15 min. The trypsin was then inactivated by adding 500 µl RPMI medium supplemented with 10% FBS. The samples were transferred to 1.5-ml tubes and centrifuged for 10 min (500 × *g*), the supernatant was removed, and the pellet was resuspended in 50 µl Cytofix solution. The fixed sample was resuspended in 200 µl 1× phosphate-buffered saline (PBS) (Fisher Scientific, Fair Lawn, NJ) and filtered using a 5-ml Falcon polystyrene round-bottom tube with a 35-µm nylon mesh cell-strainer cap (Corning Inc., Corning, NY). The filtered samples were analysed using flow cytometry (SE500 Flow Cytometer, Stratedigm, San Jose, CA). To define sporozoite populations on infected cells, we analysed filtered sporozoites stained with CFSE, but without HCT-8 cell adherence (Fig. 1A).

### *In vitro* proliferation assay

The vital dye CFSE (Thermo Fisher Scientific, Waltham, MA) was used to monitor proliferation of the intracellular stages of *C*. *parvum*. CFSE is activated by viable cells. However, with each cycle of cell division, the fluorescence intensity decreases. Thus, proliferation was evaluated by measuring the reduction in fluorescence intensity after 16 h using flow cytometry. For these experiments, the parasites were silenced (or not) and excysted as described. After excystation, sporozoites were used to infect cultured HCT-8 cells. Basal infection was allowed for 2 h at 37 °C. The monolayer was then washed, and the medium replaced with 250 µl fresh RPMI-1640 with 10% FBS and 1X antibiotic/antimycotic solution. The infected cells were incubated at 37 °C for 16 h (before parasite egress at 19–24 h). After incubation, the cells were harvested using trypsinisation and then washed, fixed, resuspended in 1 × PBS, and analysed using flow cytometry.

### Merozoite egress assay

We evaluated the effect of silencing using an egress model. We measured the numbers of merozoites in supernatants collected between 16–19 h post-infection, which correlates with the timing of egress in our model^8^. For these experiments, we induced silencing after sporozoite infection of the HCT-8 cells. After infection, the medium was removed, and the cells were transfected with ssRNA/hAgo2 complexes and incubated at 37 °C for 16 h or 19 h. The RPMI medium was removed at 16 h, replaced with 250 µl fresh RPMI medium, and incubated for 3 h to complete the 19-h incubation period. After the incubations, the supernatant was collected, filtered, and fixed as described. After the supernatant was removed, the remaining cell monolayer was trypsinised, washed, fixed, and filtered. The supernatant and the cell monolayers were analysed separately using flow cytometry.

### Anticryptosporidial activity of ellagic acid on infected cells

To evaluate anticryptosporidial effects of ellagic acid, HCT-8 cells were infected with CFSE-labelled sporozoites. After a 1-h infection, the medium was replaced with 250 µl serum-free RPMI medium (free serum) containing varying concentrations of ellagic acid (0, 3 nM, 30 nM, 300 nM, 3 µM, 7.5 µM, 15 µM and 30 µM) and each sample was incubated for 16 h. After incubation, the monolayers in the plate were washed using PBS. The cells were trypsinised, fixed, and analysed using flow cytometry as described. For the RT-PCR experiments, the monolayers were washed using PBS. After removing the supernatant, 350 µl RLT buffer was added and the RNA was extracted using an RNAeasy kit.

### Statistical analysis

For all RT-PCR experiments Sigmaplot V12 was used for the statistical analysis. Data were analyzed using unpaired two-tailed t test and presented as means ± SD. *P* < 0.05 was considered to be statistically significant.

## Results

### Actin, Rom1, NDK, and Ap2 silencing

Four, 21-nt ssRNA antisense sequences each for Actin, Rom1, DKN, and Ap2 were synthesised complementary to mRNA (Supplementary Table S2). Complexes for the four targeted genes resulted in >75% decreased expression compared with the controls (unrelated ssRNA, scramble ssRNA, or untreated parasites) (Table 1). ssRNA-Ago did not affect expression of non-targeted GAPDH mRNA, ribosomal r18s, or parasite viability (Supplementary Fig. S2).

### Silenced targets are not involved in excystation of cryptosporidium parasites

To evaluate the role of silenced genes during excystation of *Cryptosporidium* sporozoites, measured the excystation rate using fluorescence (Fig. 1A). Silencing did not result in significant reduction in excystation rate for any of the targets (Fig. 1B).

### Gene silencing of selected genes blocks parasite entry

**Figure 2:**
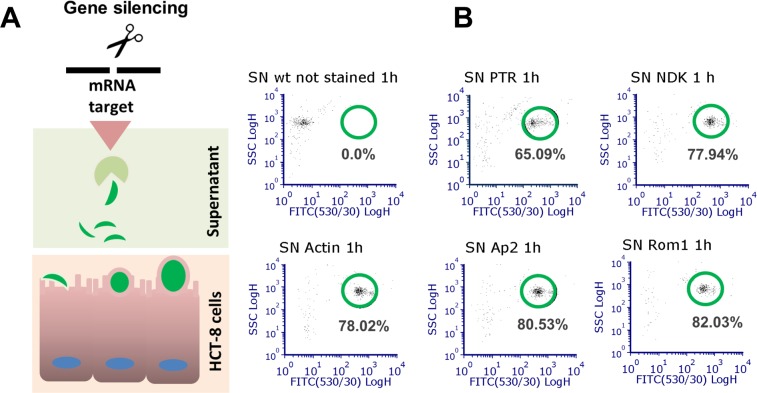
*Cryptosporidium* invasion assay. *Invasion model*. (**A**) Transfected parasites were used to infect HCT-8 cells (infected cells, red square). After a 1-h infection, supernatant (square green) was collected and sporozoite percentages were evaluated using flow cytometry. *Effect of silencing on parasite entrance using flow cytometry*. (**B**) Silencing was induced or not (PTR), and then sporozoites were used to infect cells. Parasites that did not invade cells were evaluated in the supernatants (SN). WT = unstained wild-type, PTR = parasites treated only with protein transfection reagent.

We evaluated gene silencing using an invasion model and flow cytometry (Fig. 2A). We measured the proportions of CSFE-labelled parasites that failed to invade HCT-8 cells (Fig. 2A). First, we defined sporozoite populations using flow cytometry (Supplementary Fig. S1A). For the invasion model, we transfected parasites and then evaluated the numbers of sporozoites in the supernatants. The results indicated that the control group transfected only with PTR consisted of approximately 65% of stained cells (merozoites). In contrast, silencing of Rom1 significantly increased the numbers of gated cells. This result indicated that sporozoites did not invade the host cells (Fig. 2B). Silencing of NDK, Ap2, and Actin also resulted in a partial effect on sporozoite invasion (Fig. 2B).

### NDK inhibition reduces parasite proliferation

**Figure 3:**
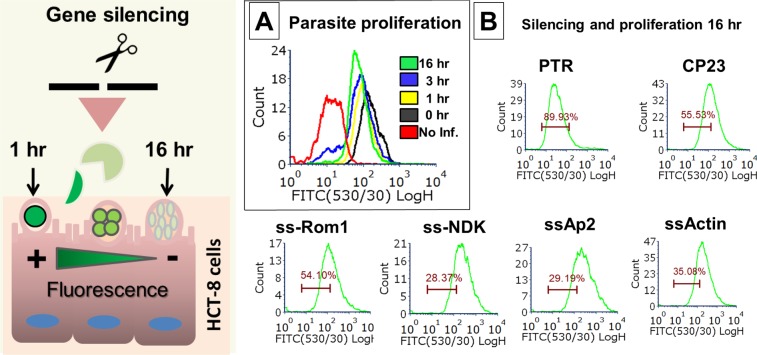
*Cryptosporidium* proliferation assay. Stained parasites (green) were transfected and then used to infect HCT-8 cells. Division of wild-type (WT) parasites within infected cells was analysed using flow cytometry (0–16 h) to monitor reductions in the fluorescent signals. (**A**) Division of the transfected parasites was analysed at 16 h and compared with untreated parasites (PTR) or parasites treated with unrelated target Cp23 (**B**).

To evaluate the effects of gene silencing on parasite division, we used a proliferation model (Fig. 3). Parasites were labelled with CFSE and collected at 16 h post-infection, before the time of egress (Fig. 3). The results of the proliferation model indicated that for controls, cell proliferation resulted in a decreased CFSE signal; 89% of cells had a decreased signal by 16 h (Fig. 3A). After silencing NKD, Ap2, or Actin, 28–35% of cells had a decreased CFSE signal (Fig. 3B). Silencing CP23 and Rom1 resulted in intermediate values (54–55%), which suggested that proliferation was partially inhibited. We also tested the effect of ellagic acid (EA) on sporozoite viability and found no EA-associated sporozoite killing effect (Supplementary Fig. S1B).

### Silencing Rom1 and AP2 reduced parasite egress

**Figure 4:**
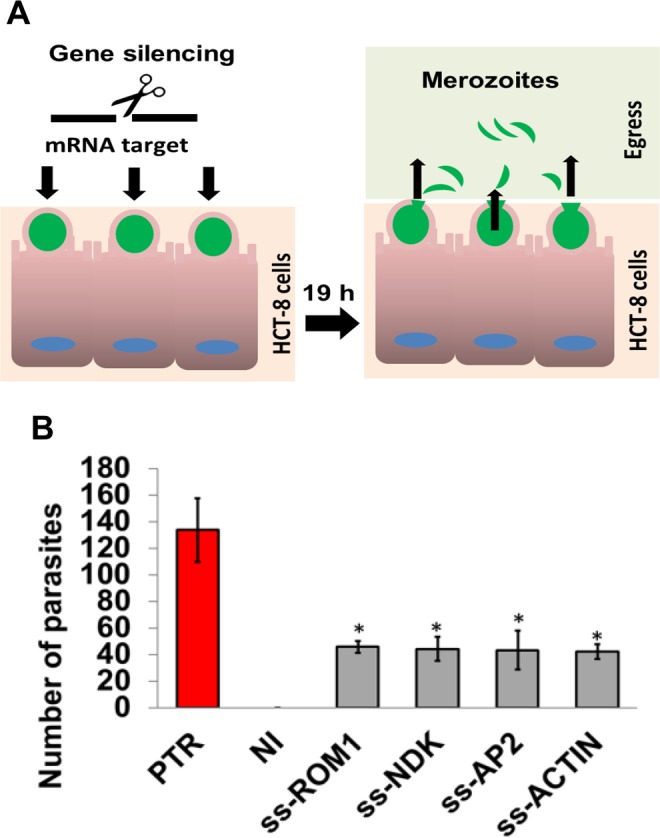
*Cryptosporidium* egress assay. Egress model. (**A**) Parasites were stained (green) and used to infect HCT-8 cells. We induced silencing in parasites within infected cells. Then, supernatants from 16–19 h were collected and evaluated using flow cytometry. (**B**) The total numbers of parasites (in 200 µl) were determined using qRT-PCR in treated samples (grey bars) and in control samples treated only with PTR (red bar) and in non-infected cells (NI). The experiments were conducted in triplicate, standard deviation (SD) values and p-values (*p ≤ 0.05) are shown.

To test the effects of silencing on egress, we transfected intracellular parasites on infected HCT-8 cells. Transfected complexes did not affect HCT-8 cell viability (Supplementary Fig. S2). However, we found a reduction in expression for all tested targets (Supplementary Fig. S3). After confirmation of silencing, fresh medium was added to collect merozoites released between 16–19 h (Supplementary Fig. S4). To evaluate the egress, we performed qRT-PCR to quantify the relative numbers of merozoites in the supernatants of treated and untreated samples (Fig. 4B). There was a significant reduction in the numbers of merozoites found in the supernatants of the silenced samples (Fig. 4B). These results indicated that silencing DKN and Actin reduced parasite proliferation and egress. Silencing Ap2 and Rom1 markedly reduced egress disproportionately to the effects on proliferation.

### Ellagic acid treatment blocks parasite proliferation

**Figure 5:**
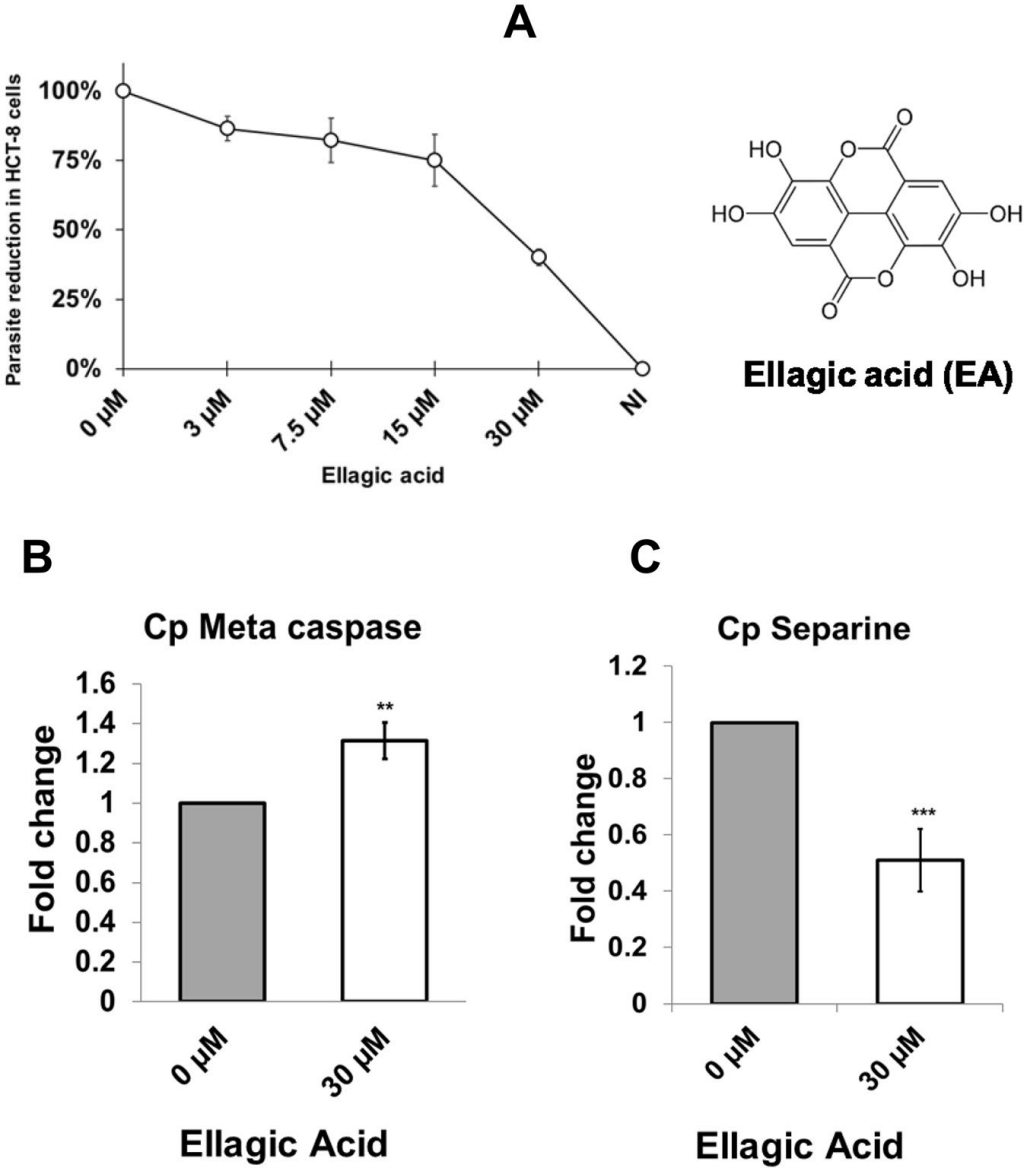
Anticryptosporidial activity of ellagic acid (EA). Reduction of parasites on infected cells using EA. (**A**) We infected human intestinal cells (HCT-8) with *Cryptosporidium*. After infection, the cells were treated with different amounts of EA and then we evaluated the parasite burden using qRT-PCR. Activation of apoptosis by EA. (**B**) We evaluated expression of the apoptosis marker metacaspase in treated (white bar) and untreated (grey bar) cells. Inhibition of proliferation by EA. Expression of the proliferation marker separine, (**C**) infected cells were treated (white bar) or untreated (grey bar) with EA, evaluated using qRT-PCR. RT-PCR experiments were performed in triplicate, and the standard deviations are indicated using bars.

Because NDK silencing had the greatest reduction on proliferation, we evaluated the anticryptosporidial activity of the NDK inhibitor EA in the HCT-8 infection model. The results indicated that EA inhibited parasite proliferation at micromolar concentrations (Fig. 5); the EC50 values were 15–30 µM. Minimal or no inhibition was noted at concentrations (0, 3, 30, or 300 nM). EA was not cytotoxic to host cells even at 30 µM (Supplementary Fig. S5). To evaluate the anticryptosporidial mechanism, we examined parasite expression of proliferation and apoptosis markers. We found significant down-regulation of separine and meta-caspases (Fig. 5B,C), which suggested that parasitostatic and parasiticidal effects occurred via of blocking of proliferation and induction of apoptosis.

## Discussion

*Cryptosporidium* lacks the machinery involved with mammalian gene silencing^9^. Our previous studies revealed the feasibility of silencing *Cryptosporidium* genes by transfecting oocysts with human Argonaute (with slicer activity) loaded with ssRNA^5^. The results of these initial experiments confirmed protein-level reduction and indicated that the method could be used to evaluate parasite invasion. Because the silencing is maintained up to 24 h, we hypothesised that this method could be used to evaluate other key biological processes during the asexual cycle of parasites maintained in HCT-8 cells (e.g., excystation, proliferation, and egress). Thus, the goal of this study was to use the silencing method to identify targets that are critical for different stages in the parasite’s lifecycle. Our first objective in this work was to identify drug-susceptible candidates for gene silencing. We used transcriptional data to prioritise genes highly expressed during invasion, proliferation, and egress. We also prioritised genes with low homology with host molecules, but that were highly conserved between *Cryptosporidium* species. After the initial analysis, we identified 100 potential candidates (Supplementary Fig. S1). We developed antisense ssRNA sequences to silence selected genes. In these experiments, the silencing rates were 30–94% (data not shown). Because partial silencing (30–75%) may not be optimal for phenotypic studies, we only used antisense ssRNA that induced a > 75% response. The genes selected for silencing included: (1) Actin, an structure essential for *Cryptosporidium* motility^10^, (2) NDK, an essential protein for synthesis of nucleotides^11^, (3) Rom1, a protease involved in invasion and egress in other apicomplexans^12^, and (4) Ap2, which is a transcription factor involved in proliferation^10^. First, we evaluated the effect of silencing during parasite excystation. The excystation assays revealed that none of the silenced genes affected this process, which suggested that these proteins are not essential for excystation. This result was expected, because transcriptomic studies found that most genes (approximately 85%) in *Cryptosporidium* are expressed after the excystation process^13^. The invasion assay results indicated that silencing of Rom1 blocked parasite entry. This proteolytic enzyme has been implicated in parasite invasion^14^. Orthologue rhomboid protease in *Toxoplasma* cleaves cell surface adhesins; this protein is essential for invasion^14^. In *Plasmodium*, PfRom1 and PfRom4 assist with merozoite invasion by catalysing the intramembrane cleavage of the merozoite adhesin AMA1^14^. Actin silencing also resulted in inhibited invasion. Apicomplexan parasites actively invade host cells using a mechanism likely to be powered by a parasite actin-dependent myosin motor. Actin involvement in invasion was first suggested by study results that revealed the ability of the actin polymerisation inhibitor cytochalasin D to block invasion^15^. The proliferation assay results indicated that NDK, Ap2, and Actin, but not Rom1, reduced parasite proliferation. NDKs are enzymes required for the synthesis of nucleoside triphosphates (NTPs), other than ATP. They provide NTPs for nucleic acid synthesis, CTP for lipid synthesis, UTP for polysaccharide synthesis, and GTP for protein elongation, signal transduction, and microtubule polymerisation. NDK is essential for intracellular parasite proliferation. Actin proteins are associated with cytoskeleton motility during cell division. *Cryptosporidium* transcriptomic analysis found that actin is highly expressed between 12–48 h after infection; during this time the parasite is actively dividing, from a single cell (trophozoite) to eight cells (meronts II) in 24 h. We found that silencing of Ap2 transcription factor also affected proliferation (Fig. 3B). Ap2 proteins are transcription factors that harbour a plant-like DNA-binding domain. Five Ap2 proteins have been identified as key stage-specific regulators in *Plasmodium*. Thus, Ap2 proteins have been implicated in *P*. *falciparum* var gene regulation; they bind the SPE2 DNA motif and act as a DNA-tethering protein involved in formation and maintenance of heterochromatin^16^. The roles of Ap2 proteins in gene regulation have also been investigated in *T*. *gondii*; results suggest that some Ap2 proteins regulate progression through the cell cycle^17^ and crucial virulence factors^18^. Other study results suggest that Ap2s regulate developmental transition^19^. Radke *et al*.^20^ characterised a *T*. *gondii* Ap2 and found that this molecule acts as a repressor of bradyzoite development. Our results indicated that the egress assay was partially affected by parasite proliferation (Fig. 4B). Silencing of NDK, Ap2, and Actin blocked proliferation, leading to a reduction in the number of merozoites (Fig. 3B). In contrast, silencing with Rom1 had an even greater effect on egress. Because that protein only had moderate effects on proliferation (Fig. 3B), it is likely that Rom1 affects parasite egress via a proteolytic mechanism. In *Plasmodium*, release of merozoites from schizonts result in the movement of *Plasmodium* Rom1 from the lateral asymmetric localisation to the merozoite apical pole and the posterior pole^21^.

Overall, the results of our *in vitro* studies indicated that silenced genes block proliferation and egress in *Cryptosporidium* parasites. Therefore, we hypothesised that chemical inhibitors against these enzymes should arrest *Cryptosporidium* proliferation in infected cells. Because silencing of NDK resulted in effects during invasion, proliferation, and egress, it was selected for further studies. The inhibition of NDK activity by EA has been demonstrated^22,23^. Thus, here we tested EA using the *Cryptosporidium* infection model. We observed the anti-cryptosporidial activity of this compound at micromolar concentrations (Fig. 5A). In preliminary studies, we have observed >2 log differential inhibition comparing semi purified *Cryptosporidium* NDK vs human NDK (data not shown). EA is a natural compound found in strawberries and other fruits; thus this compound has been used as a dietary complement to treat disease^24,25^. EA has antimicrobial activity against the gastrointestinal pathogen, *Helicobacter pylori*^26^. To investigate the mechanism of anticryptosporidial activity, we evaluated expression proliferation (separine) and apoptosis markers (metacaspase) in the parasite. We found a down-regulation of separine expression (also known as separase), which is implicated in chromatin regulation during meiosis and mitosis processes^27^. This finding suggested that EA may be blocking proliferation through NDK inhibition, as found in the silencing experiments. However, we also found an up-regulation of metacaspase, which suggested that other mechanisms may be involved in parasite killing. EA has multiple benefits for human health through enhancement of immune system or epithelial barriers^28–30^. Thus, we speculate that it has a dual effect on *Cryptosporidium* by reducing the infection through the activation of host pathways (e.g., defensin secretion) and affecting essential enzymes in parasites. The micromolar concentrations tested here were less than the biological concentrations of EA acid commonly used in humans^31^. Therefore, future studies will be focused to characterise the effects of EA and metabolites on intestinal cells of infected mice. If the results of these studies support the findings of anticryptosporidial activity and activation of host response, then we anticipate that this compound could serve as a lead compound for treatment in humans infected with *Cryptosporidium*.

## Supplementary information

Supplementary data

## Data Availability

All the other data supporting the findings of this study are available within the article and its supplementary information files and from the corresponding author upon reasonable request. A reporting summary for this article is available as a Supplementary Information file.
