# Supplementary material for: Systematic gene silencing identified Cryptosporidium nucleoside diphosphate kinase and other molecules as targets for suppression of parasite proliferation in human intestinal cells
Source: Sci Rep. 2019 Aug 21;9:12153. doi: 10.1038/s41598-019-48544-z (PMC6704102; doi:10.1038/s41598-019-48544-z)

**Systematic gene silencing identified *Cryptosporidium* nucleoside diphosphate kinase and other molecules as targets for suppression of parasite proliferation in human intestinal cells**

Castellanos-Gonzalez A^1*^, Martinez-Traverso G^1^, Fishbeck K^1^, Nava S^1^, White AC Jr^1^

Infectious Disease Division, Department of Internal Medicine, University of Texas Medical Branch, Galveston, TX^1^

***Address correspondence to:**

Alejandro Castellanos-Gonzalez

Infectious Disease Division

Department of Internal Medicine

University of Texas Medical Branch

301 University Boulevard, Route 0435

Galveston, TX 77555-0435

Email: alcastel@utmb.edu

**Legends for Supplementary Tables and Figures**

**Supplementary Table S1.** Accession numbers of *Cryptosporidium* sequences used for antisense ssRNA design.

**Supplementary Table S2.** *Cryptosporidium* antisense ssRNA sequences used in this study.

**Supplementary Table S3.** Target genes and primer sequences used for RT-PCR analysis.

**Supplementary Figure S1.** Sporozoites of *Cryptosporidium*, flow cytometry results (A). Cryptosporidium oocysts were stained with CFSE and then excystation was induced. Parasites were filtered and then sample was analysed using flow cytometry. Effects of EA on oocysts and excystation analysed using flow cytometry (B), we treated stained oocysts with EA and then excystation was induced.

**Supplementary Figure S2.** Expression of the human 18s gene in HCT-8-cells after transfection. HCT-8 cells were infected with *Cryptosporidium* sporozoites and then transfected with ssRNA. After a 2-h infection period, the supernatant was removed, and new culture medium was added. Silencer complexes were then added to the infected cells and incubated overnight. To evaluate cytotoxic effects of ssRNA or HCT-8, we quantified 18s rRNA. Cell death indicated reduction of this marker. The figure indicates there were no differences between wild-type (WT), protein transfection reagent alone (PTR), and the tested ssRNAs ssAct, ssAp2, ssNDK, and ssRom1 (encapsulated in PTR).

**Supplementary Figure S3.** Gene silencing after infection. We examined silencing on intracellular forms (merozoites) within HCT-8 cells. We infected and treated cells and evaluated silencing using qRT-PCR in total RNA from infected cells transfected with silencer complexes. The figure shows PCR CT values from duplicate experiments of transfected cells with silencer complexes (grey bars) and cells transfected only with PTR. All cells treated with ssRNA showed had amplification cycle delays, compared with the control cells. All samples were normalised against GAPDH. The standard deviations for the PCR triplicates are indicated using bars.

**Supplementary Figure S4.** *Cryptosporidium* merozoites were collected at 16 h after infection. *Cryptosporidium* merozoites analysed using flow cytometry. HCT-8 cells were infected with labelled sporozoites. After infection, silencing was induced, and at 16 h after infection (before egress) supernatant was removed and fresh medium was added. Supernatant was collected again at 19 h and analysed using flow cytometry. The percentages (%) of merozoites were evaluated in samples treated only with PTR (left); merozoites were not found in supernatants of non-infected samples (right).

**Supplementary Figure S5.** Ellagic acid and viability of HCT-8 cells. Effect of EA on non-infected (NI) HCT-8 cells. HCT-8 cells were treated with EA (or not) and then viability was evaluated using measurement of the activation of the vital dye CFSE. The figure shows the percentages (%) of cells positives for CFSE.

Table S1.

| **Target Name** | **Accession Number** |
| --- | --- |
| Signal peptide plus thr stretch, cys rich, possible Mucin | XM_627858 |
| Heat shock protein 90 | XM_626924 |
| ATP synthase beta chain | XM_001388163 |
| Translation initiation factor if-5A | XM_628401 |
| 60S Ribosomal protein L37A | XM_626792 |
| Possible low mw mucin glycoprotein locus of 6 genes | XM_625409 |
| Lactate dehydrogenase | XM_628236 |
| Peter Pan like protein | XM_627234 |
| Protein Phosphatase 2C family protein phosphatase | XM_628610 |
| Periodic Tryptophan protein 1 (PWP1) family protein | XM_625860 |
| Adenosylhomocysteinase | AY161083 |
| Inosine-5-monophosphate dehydrogenase | XM_625342 |
| Thrombospondin related adhesive protein | AF017267 |
| Cryptosporidium parvum antigen, CP2 | AY471868 |
| Cryptopain 1 | DQ156545 |
| Bifunctional Dihydrofolate reductase-thymidylate synthase | U41366 |
| Phospholipase C domain | XM_625844 |
| Glutathione peroxidase | XM_626631 |
| Glutaredoxin related protein | XM_626456 |
| Superoxide dismutase | XM_001388246 |
| Thioredoxin reductase | FX115924 |
| Actin | FX115681 |
| Apicomplexan hypothetical protein, Ap2 | XM_627219 |
| Nucleoside-diphosphate kinase | FX115659 |
| Hypothetical protein, possible conserved domain (ZInc finger) | XM_627320 |
| Immunodominant antigen 23393226 | XM_625938 |
| Fatty acid synthetase | AY380457 |
| Sporozoite antigen, Profilin | XP_001388102 |
| Sporozoite antinge, putative, Fibrinogen | FX115109 |
| Conserved hypothetical protein, Serine | FX115685 |
| TB2/DP1/HVA22 family integral membrane protein that may be involved in membrane trafficking | XM_628022 |
| Hypothetical protein with signal peptide | XM_627729 |
| Myosin | XM_625764 |
| Triosephosphate isomerase | XM_628120 |
| Glyceraldehyde 3 phosphate dehydrogenase | XM_627718 |
| 6 phosphate glucose isomerase | XM_626511 |
| Phosphatidylinositol 4-kinase | XM_627336 |
| Hypothetical protein, rhomboid 1 | FX115596 |
| Rhomboid family membrane associated protease, 7 transmembrane domain | XM_628467 |
| Hexokinase | XM_627719 |
| Fructose-1,6-bisphosphate aldolase | XM_628118 |
| Calcium-dependent protein kinase 1 | AY466385 |
| Calcium-dependent protein kinase 2 | AY466386 |
| Calcium-dependent protein kinase 3 | AY466387 |
| Calcium/calmodulin-dependent protein kinase with a kinase domain and 4 calmodulin like EF hands | XM_628359 |
| Calcium-dependent protein kinase 7 | XM_662940 |
| cAMP-dependent protein kinase regulatory subunit | XM_001388325 |
| Casein kinase I partial | XM_001388066 |
| cGMP-dependent protein kinase gene | AF413571 |
| Mitogen-activated protein kinase 1 | XM_001388178 |
| Phosphatidylinositol-4-phosphate 5-kinase | XM_001388398 |
| Protein kinase, putative (cgd2_3190) | XM_001388195 |
| Protein kinase, cAMP-dependent, catalytic chain | XM_001388082 |
| Mitogen-activated protein kinase 2, putative | XM_001388209 |
| RIO-like kinase domain; N-terminal region conserved | XM_628232 |
| Protein kinase domain, putative (cgd2_1610) | XM_001388165 |
| Ser/Thr protein kinase (cgd5_3180) | XM_626246 |
| Ser/Thr protein kinase (cgd5_4390) | XM_625601 |
| Casein kinase II, alpha subunit, putative | XM_001388265 |
| Serine/threonine kinase-1 (cgd5_250) | XM_001388212 |
| Serine/threonine-protein kinase, putative (cgd6_4960) | XM_001388318 |
| Cryptopain - cysteine proteinase secreted | XM_627814 |
| Ubiquitin carboxyl-terminal hydrolase of the cysteine proteinase fold | XM_627322 |
| Aspartyl (acid) protease, putative | XM_001388146 |
| NAD dependent dehydrogenase | XM_627076 |
| NAD kinase | XM_627101 |
| e3 ubiquitin-protein ligase, putative | XM_001388289 |
| FKBP isomerase | XM_628213 |
| phenylalanyl-tRNA synthetase beta chain | XM_627227 |
| putative phenylalanyl-tRNA synthetase | XM_626883 |
| GMP synthase | XM_625300 |
| MJ1157-like thiouridine synthase (Pploop atpase) plus Zn ribbon | XM_626012 |
| Acetyl-CoA carboxylase like biotin dependent carboxylase | XM_627260 |
| RPR46-like RNAse PH domain | XM_625789 |
| RNAse P Rpr2/Rpp21 subunit domain | XM_627476 |
| Type I fatty acid synthase | XM_626786 |
| Serine/threonine protein kinase; possibly sporulation specific | XM_663517 |
| Cellular apoptosis susceptibility gene | XM_662229 |
| Apoptosis-related protein | XM_663128 |
| Calpain like thiol protease | XM_627039 |
| Glycosylphosphatidylinositol transamidase, involved in GPI anchor biosynthesis | XM_627883 |
| UDP N-acetylglucosamine transporter-like nucleotide sugar transporter | XM_626469 |
| DP-fucose transporter | XM_627044 |
| Zinc ZIP transporter protein | XM_627431 |
| Possible copper transporter | XM_628081 |
| UDP-galactose transporter | XM_627867 |
| Putative amino acid transporter | XM_626977 |
| P-type ATpase | XM_625857 |
| ATP-dependent helicase | XM_001388049 |
| Elongation factor-like protein | XM_661307 |
| ATP-binding cassette protein | XM_001388108 |
| Uvb-resistance protein uvr8 | XM_662805 |
| Alpha tubulin | XM_625871 |
| Tubulin beta chain | XM_627803 |
| Gamma tubulin | XM_628373 |
| Dihydrofolate reductase-thymidylate synthase | XM_625460 |
| ABC transporter protein putative | XM_001388321 |

Table S2.

| **Single interfering RNA** | **Sequence** |
| --- | --- |
| **SsCDPK1*** | 5’rCrArCrUrUrCrCrUrCrUrCrUrCrUrCrCrUrCrCrC TT-3’ |
| **ssActin*** | 5'rCrArUrArUrGrArArCrUrArCrCrArGrArUrGrGrCTT 3' |
| **ssAp2*** | 5'rGrCrUrArGrUrGrGrArUrArUrArGrGrGrArUrGrC TT3' |
| **ssNDK*** | 5'rArArArCrUrArGrCrCrCrArCrArArUrCrCrArCrGTT3’ |
| **ssRom1*** | 5'rUrArArCrGrArCrUrArCrUrUrGrArArUrGrGrG TT 3' |
| **Scrambled (Negative control)** | 5' rGrGrGrUrCrArCrGrUrGrCrGrArArCrArUrGrA 3' |

* ssRNA sequences that induce silencing >75% by RT-PCR.

Table 3

| **Target** | **Accession Number** | **Primer sequence** |
| --- | --- | --- |
| **GAPDH*** | XM_627718 | Forward: 5’-TGA CCC ATC AAA ATT CAA CG-3’  Reverse: 5’-ACG GAA ACA TCT GGA GTT GG-3’ |
| **CP23*** | XM_625938 | Forward: 5’-CAA TCA GCA ACC AAG CTC AA-3’  Reverse: 5’-TTG TTG AGC AGC AGG TTC AG-3’ |
| **CDPK1**** | XM_001388059 | Forward: 5’-GCA GTA GGG AAT ACA GGA ACA A-3’  Reverse: 5’-CCG CTA CCA CTC TGA AGA AA-3’ |
| **Actin**** | FX115681 | Forward: 5’-CAT TGC TTT AGA TTA CGA GGA AGA-3’  Reverse: 5’-TTG GAA TAA AGC CTC AGG ACA-3’ |
| **Ap2**** | XM_627219 | Forward: 5’-TAA ACA AGC CTG AGC CAA CC-3’  Reverse: 5’-TCG GTT CCA ATT TCA TGT CC-3’ |
| **NDK**** | FX115659 | Forward: 5’-GCA ACT CGA CTG AAT CGT CA-3’  Reverse: 5’-CTT TCC TGC ATG ATC GGA AT-3’ |
| **GP900***** | AF068065 | Forward: 5’-GCA CCATTT GAG TTG GAT GTT AG-3’  Reverse: 5’-AGT TGG GAG ACC AGT GAT AGA-3’ |

* Primers for housekeeping genes (non-silenced) used for normalization.

** Primers for positive ssRNA controls used for silencing.

***Primers for non-targeted gene during silencing (i.e. silencing with ssNDK does not affect GP900 expression in treated parasites vs WT)

Figure S1.

A

B


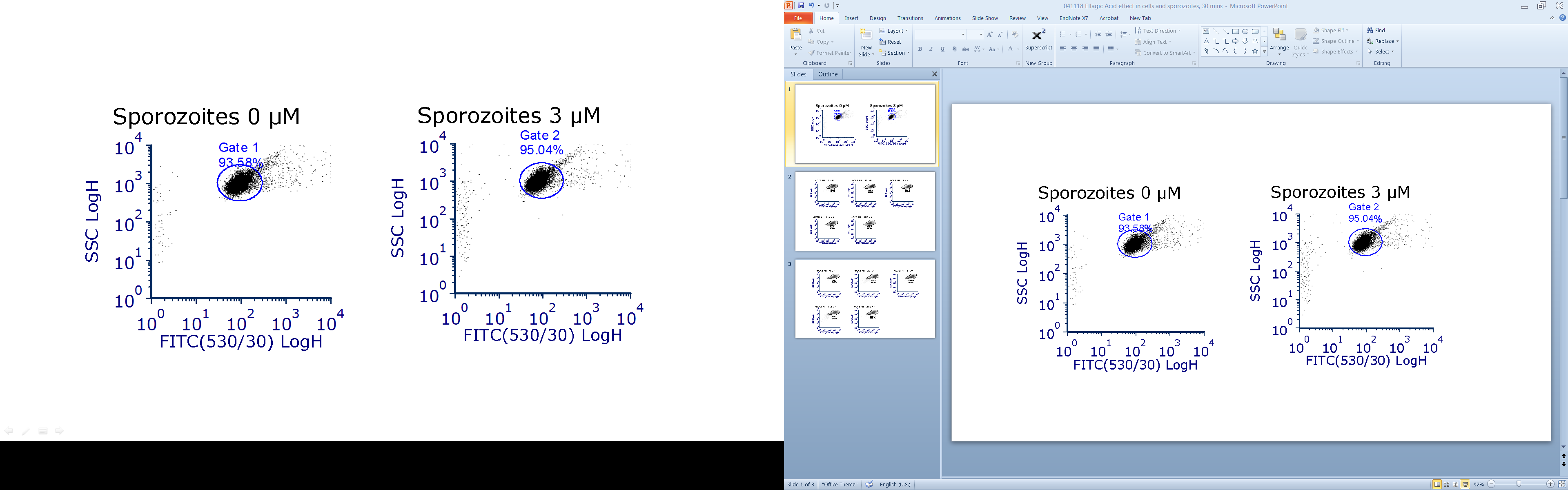


Sporozoites-CFSE WT Sporozoites-CFSE EA 15 µM

Figure S2.


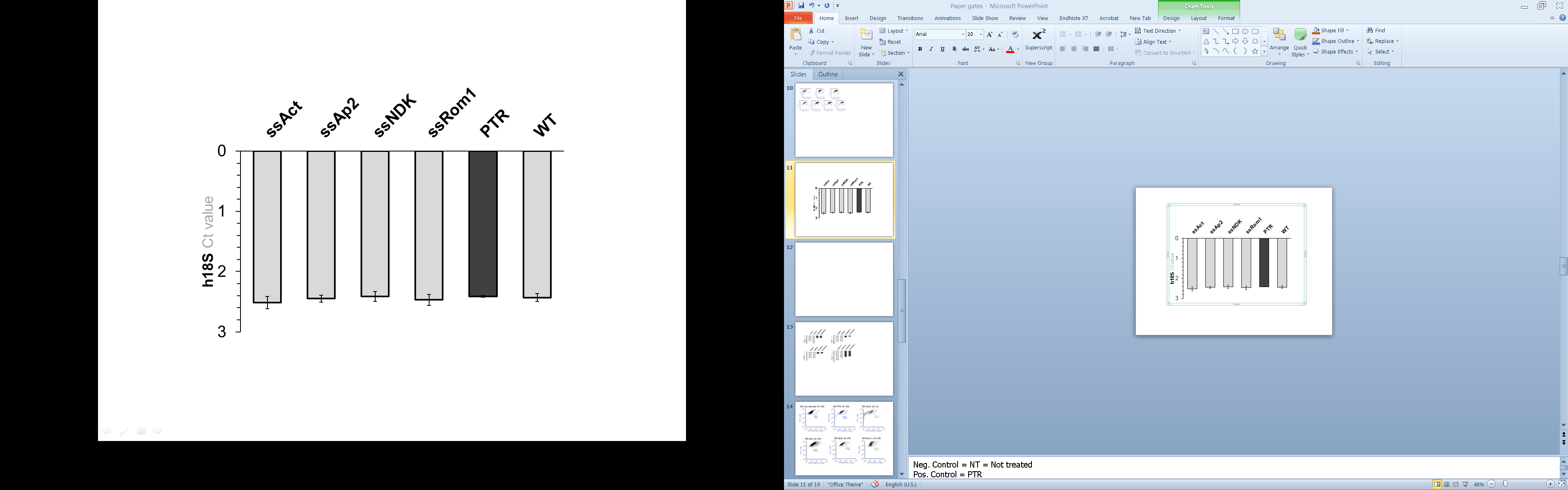


**Figure S3. Gene silencing after infection**.


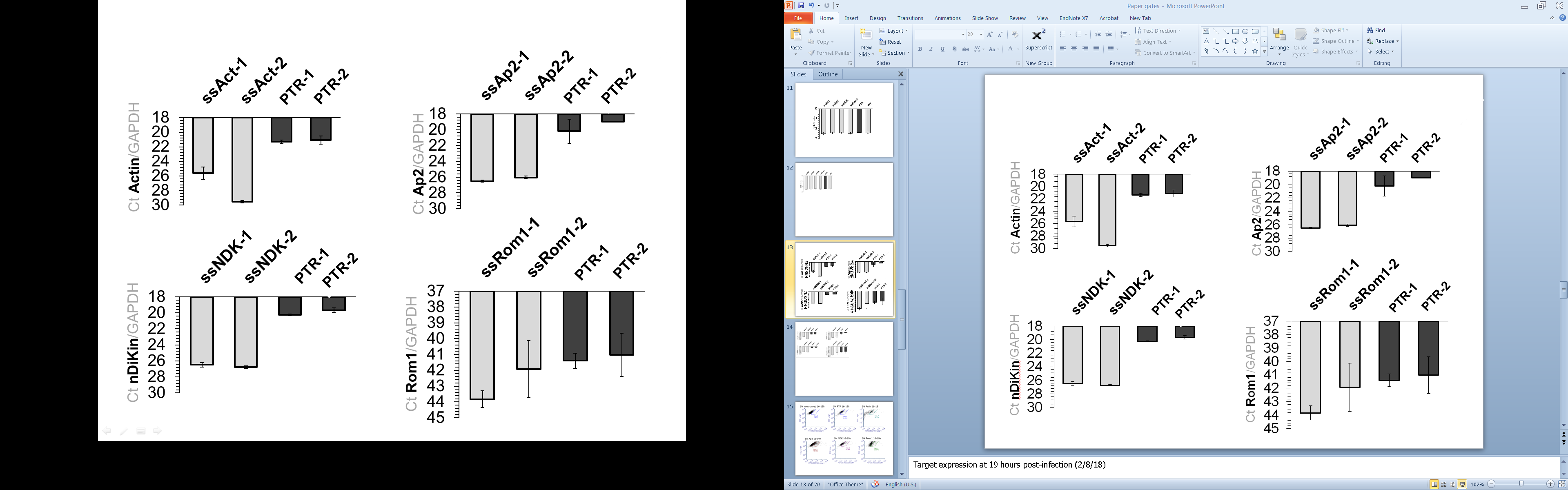


Figure S4.


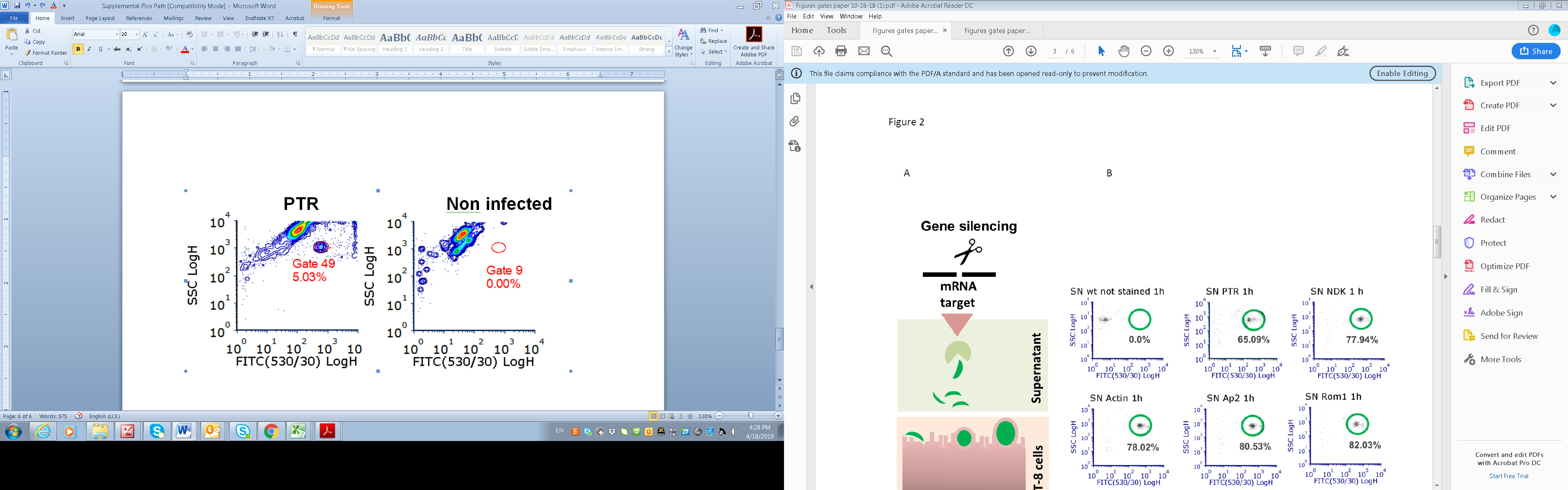


Figure S5.


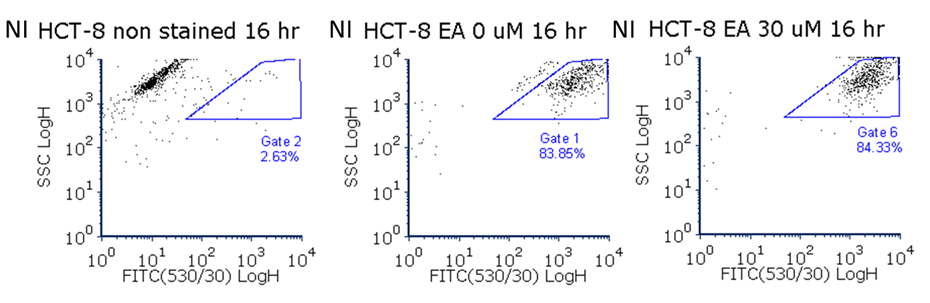

Supplement: Supplementary file 1 — Supplementary data [file 41598_2019_48544_MOESM1_ESM.doc]
